# Needle in a haystack: a case report of splenic foreign body-associated sepsis

**DOI:** 10.1093/jscr/rjab525

**Published:** 2021-11-29

**Authors:** Benedict R H Turner, James Barnacle, Hemant Sheth

**Affiliations:** Department of Surgery and Cancer, Imperial College London, London, UK; Infectious Diseases, London Northwest NHS Healthcare Trust, London, UK; Ealing Hospital, London Northwest NHS Healthcare Trust, London, UK

## Abstract

Instances of foreign bodies impacted in solid organs are rare, and rarer still are reports of objects in the spleen. A 42-year-old presented septic with abdominal pain, high inflammatory markers and haemodynamic instability. She was found to have a splenic haematoma and a 4-cm hyperdense foreign body within the spleen. Ultrasound-guided drainage of the haematoma isolated *Streptococcus anginosus* and conservative management with intravenous antibiotics avoided the need for emergency splenectomy. The bacterium isolated was the same cultured 9 months previously from the patient’s empyema fluid. The origin of the foreign body was not identified, though is made of metal and pre-dates any hospital admissions. The case raised the question of how an object might penetrate the spleen without knowledge of the patient and highlighted the risks of foreign body-associated sepsis, the risks and benefits of emergency splenectomy and management of complex cases with paucity of evidence.

## INTRODUCTION

Penetration of a solid organ by a foreign body is a well-documented phenomenon, though remains uncommon in everyday practice [1–5]. With regard to the spleen, there have been three cases of foreign body impaction reported [1, 2, 5]; all in a paediatric cohort after direct ingestion. The case hereafter is the first instance of a splenic foreign body in an adult and associated sepsis, highlighting the perils of foreign body impaction and the risk–benefit dichotomy of surgical intervention. It also raises how a foreign body might enter the spleen unbeknownst to a patient.

## CASE REPORT

A 42-year old female carer presented to the emergency department with haemodynamic instability, fevers, rigours, new oxygen requirement and supraumbilical abdominal pain for 2 days. Her background included type 2 diabetes, hypertension, obstructive sleep apnoea, morbid obesity and a previous video-assisted thoracoscopic surgery (VATS) procedure for loculated empyema 9 months previously. Examination revealed upper abdominal tenderness with voluntary guarding, but no rigidity. Record of vital signs revealed she was shocked, tachycardic and hypoxic with oxygen saturations of 92% on room air.

**Figure 1:**
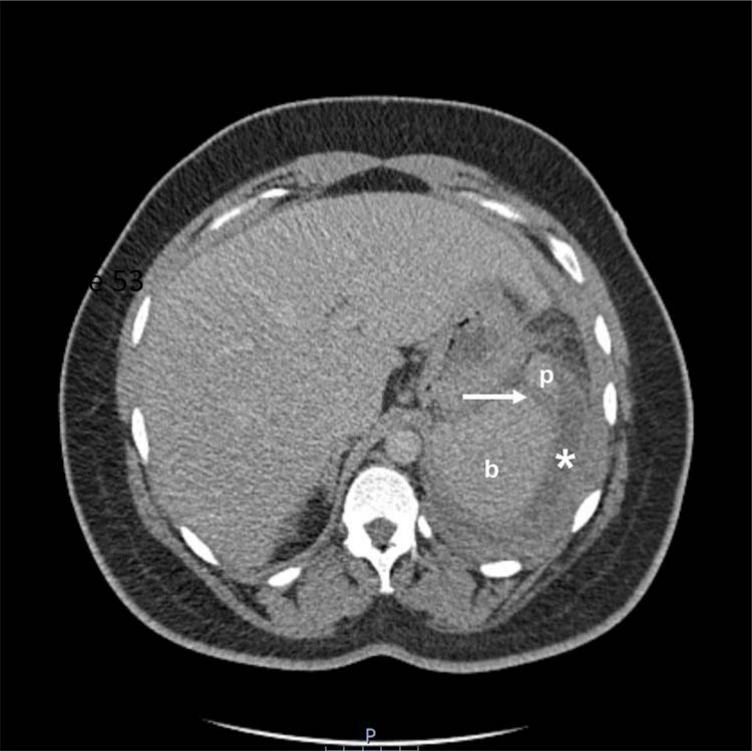
Two-mm axial section from a CT scan showing the perisplenic haematoma (*) and hypodensity (arrow) separating the upper anterior pole of the spleen (p) from the body (b). CT section number 54.

**Figure 2:**
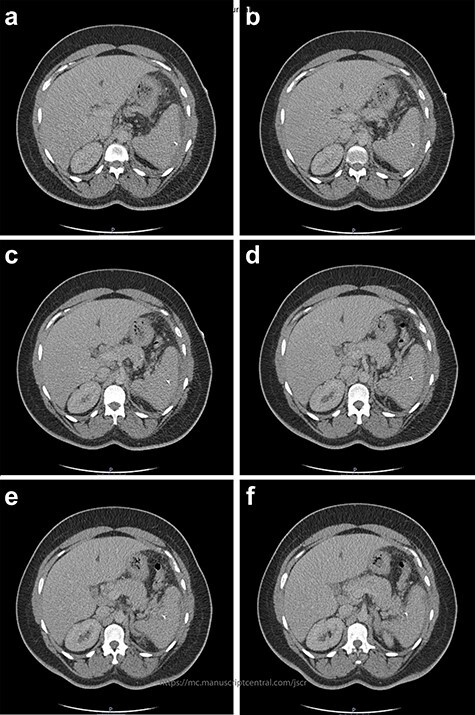
Two-mm axial sections from a CT scan demonstrating the linear hyperdensity traversing the spleen across multiple cross sections. (**a**) Section number 65 (**b**) section number 67 (**c**) section number 69 (**d**) section number 71 (**e**) section number 73 (**f**) section number 75.

**Figure 3:**
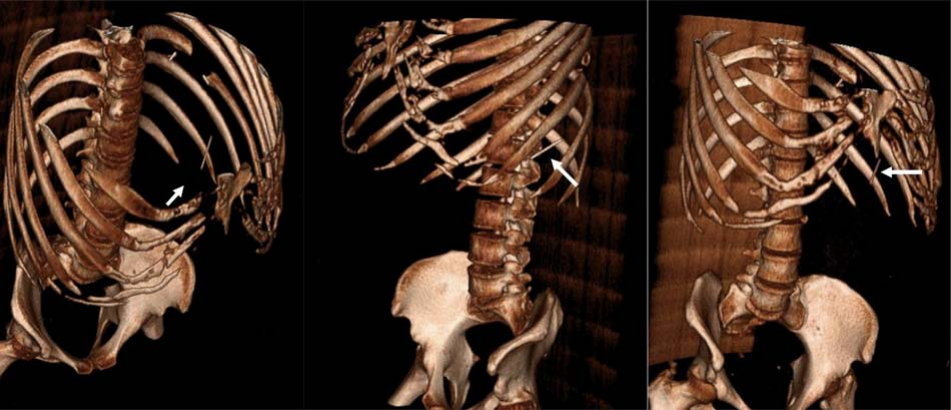
Three-dimensional reconstruction CT scan displaying the foreign body (white arrow) in three different orientations.

The initial bloods showed haemoglobin 121 g/l, white cell count 29.2x10^9^/l, lactate 0.8 mmol/l, C-reactive protein 348 mmol/l and deranged alkaline phosphatase at 175 mmol/l, with other blood results in normal ranges. A diagnosis of intra-abdominal sepsis secondary to acute cholecystitis was suspected and initial resuscitation was commenced. A computerized tomography (CT) scan of the thorax, abdomen and pelvis demonstrated a mild left pleural effusion with bibasal subsegmental consolidation, splenic subcapsular haematoma, splenic hypodensity and a linear hyperdensity density crossing the upper anterior pole of the spleen, referred to hereafter as the foreign body (Figs 1–3).

In light of a penicillin allergy, the patient was commenced on teicoplanin, ciprofloxacin and metronidazole. Attempts to drain the lung were all dry, but haematoma was successfully drained from the spleen and sent for microscopy, culture and sensitivity. The fluid grew *Streptococcus anginosus,* sensitive to teicoplanin, but the inflammatory markers failed to resolve, with c-reactive protein peaking at 548 and white cell count remaining >20 after 7 days of treatment.

**Figure 4:**
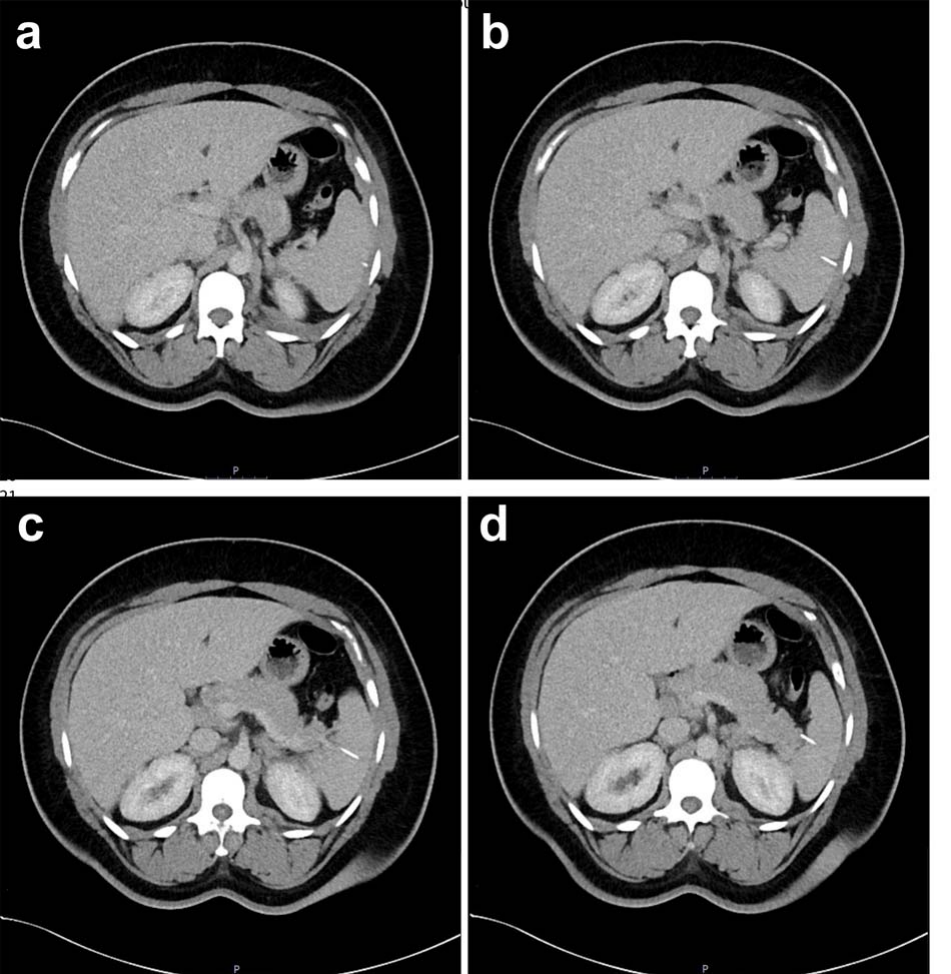
Two-mm axial sections from a CT scan in January 2020, performed after the VATS procedure, to check resolution of the empyema that shows the foreign body impacted in the spleen without evidence of haematoma. (**a**) section number 18, (**b**) section number 19, (**c**) section number 20, (**d**) section number 21.

Contact with the previous hospital that treated the patient for her empyema revealed the splenic foreign body pre-dated any hospital admission or intervention; the empyema fluid culture had grown *Staphylococcus aureus* and *S. anginosus*, and that there was no splenic collection on CT before the VATS procedure (Fig. 4). The patient had no previous hospital admissions, operations or other exposure to needles; the GP confirmed that there was no past psychiatric history. The patient had moved to the UK from Grenada, Caribbean aged 7 and that she was not aware of any procedures prior to this age.

**Figure 5:**
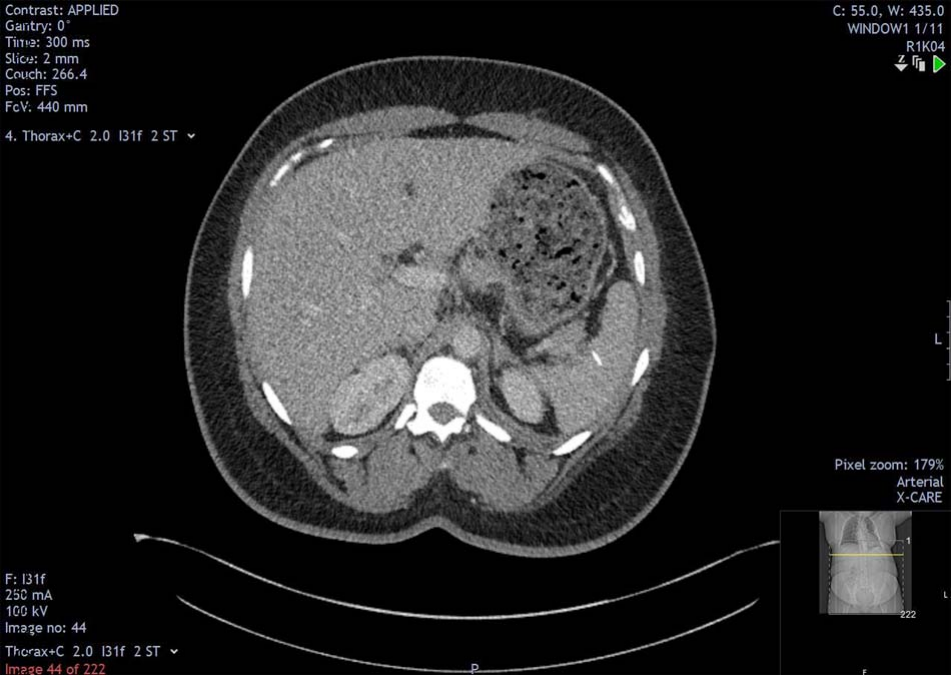
Two-mm axial section from the outpatient CT scan performed in November 2021, demonstrating resolution of the perisplenic haematoma with foreign body *in situ.*

The patient remained clinically unwell and was considered for emergency splenectomy, with discussion in a multidisciplinary meeting. Fortunately, on Day 10 of treatment, a sustained decreased in inflammatory markers and resolution of the patient’s abdominal pain led to the multidisciplinary decision that the patient would be treated conservatively with intravenous antibiotics for 3 weeks. Once medically stable, the patient was placed on a further 3-week course of linezolid and booked for a follow-up outpatient CT with review in clinic. The scan showed resolution of the perisplenic collection, with the foreign body *in situ* (Fig. 5). The plan was to pre-vaccinate the patient in case of future emergency surgery; however, the patient did not attend the follow-up clinic appointments.

## DISCUSSION

This case presented a number of diagnostic uncertainties and management dilemmas. Definite facts ascertained about the foreign body were that it was at least the same density as bone, ~4 cm in length and was not hollow. From this, it was speculated that the object might be a needle, as seen in other cases [3, 4]. There are two manners of foreign body impaction in the spleen, transgastroenteric or transcutaneous [1–5], neither of which appeared feasible here and the lack of any history of foreign body ingestion, psychiatric history nor prior procedures is perplexing. The absence of reactionary inflammation at the time of presentation implicates its presence in the spleen for a protracted, perhaps since childhood. Haematoma occurrence after the VATS procedure may indicate that perioperative seeding was responsible for the foreign body colonization.

*Streptococcus anginosus* is found in the normal flora of the pharynx, gastrointestinal and genitourinary tract. It has a propensity for abscess formation and is particularly associated with skin and soft tissue, intra-abdominal, solid organ and chest infections [6, 7]. Approximately half of patients have co-morbidities, with 25–33% of patients being diabetic in two large retrospective studies [6, 7]. In this case, deep-seated infection elsewhere was ruled out with echocardiography, orthopantomogram and CT thorax, abdomen and pelvis pointing to the empyema as the most likely source, via direct transdiaphragmatic or bacteraemic spread. Both dissemination phenomena have been noted with *S. anginosus* and a previous report documents a case of sympathetic empyema following splenic abscess [8, 9].

Given this patient’s surgical risk factors (intra-abdominal sepsis, obesity, obstructive sleep apnoea, type 2 diabetes), she was considered a poor surgical candidate. Moreover, splenectomy increases susceptibility to encapsulated bacterial infection; thus, lifelong phenoxymethylpenicillin prophylaxis is recommended, which was precluded here by penicillin allergy. Despite prophylaxis, patients undergoing traumatic splenectomy have higher risk of hospitalization and mortality from pneumonia, meningitis, sepsis, venous thromboembolism and malignancy [10]. Because of this, there has been a paradigm shift away from splenectomy and towards more conservative surgical approaches in the 21st century with excellent outcomes [11], and this case perfectly highlights the risk–benefit balance that must be contemplated before operating.

Practising evidence-based medicine means combining best research evidence, clinical judgement and patient values [12]. In absence of literature, we are most grateful to the general surgery, radiology, respiratory and infectious diseases team at our centre for their clinical judgement. Foreign body-associated infections are difficult to treat and require a multidisciplinary approach to deliver optimal care. The origin of the foreign body in this case remains a mystery and the authors invite comments on its nature from other clinicians.
